# Editorial

**Published:** 1881-06

**Authors:** 


### Editorial.

*We present to our readers with this number of the CHICAGO MEDICAL JOURNAL AND EXAMINER, a supplement of 100 pages, in which will be found a full report of the proceedings of the late meeting of the American Medical Association, collated in part from the daily edition of the Virginia Medical Monthly, the Editors of which deserve much credit for their efforts to publish daily full reports. This large addition to the cost of the Journal for the year and to the number of pages in the annual volume, is gained without any increase in the price of the Journal to our subscribers. The report, it is believed, is more full than any which has yet appeared, and we trust that this enterprise on the part of the editors and publishers will receive the appreciation which it is aimed to secure. We beg leave to remind our friends at this time, also, that we expect soon to present them with an excellent report of the transactions of the International Medical Congress, and that in our next issue we expect to report the proceedings of the Illinois State Medical Society, which is now in session in this city. No pains nor expense will be spared, in the future as in the past, to keep this Journal of Medicine in the forefront of all enterprises having in view the advancement of the interests of the profession.*

